# Three-dimensional transesophageal echocardiography in diagnosis of intermediate atrioventricular septal defect in the adult: case report and literature review

**DOI:** 10.1186/s13019-021-01596-7

**Published:** 2021-07-30

**Authors:** Ingrid Schusterova, Marta Jakubova, Marianna Vachalcova, Karolinska Sieradzka, Claudia Gibarty, Tibor Poruban, Ivo Gasparovic, Panagiotis Artemiou

**Affiliations:** 1 Medical Faculty of University Pavel Jozef Safarik, First Cardiology Clinic, Eastern Slovakian Institute of Cardiovascular Diseases, Kosice, Slovakia; 2 Medical Faculty of the Comenious University, Clinic of Cardiac Surgery, National Institute of Cardiovascular Diseases, Pod Krasnou horkou 1, 83101 Bratislava, Slovakia

**Keywords:** Intermediate atrioventricular septal defect, Transesophageal echocardiography, Tranthoracic echocardiography, 2D-echocardiography, 3D-echocardiography

## Abstract

**Background:**

Intermediate type atrioventricular septal defect is less frequent than complete or partial atrioventricular septal defect, and is rarely encountered in the elderly and the utility of three dimensional transesophageal echocardiography in the diagnosis has not been reported to date.

**Case presentation:**

In this case report, we described a rare case of an intermediate atrioventricular septal defect in an adult patient and we showed the valuable utility of real time 3D transesophageal echocardiography in the diagnosis and future surgical planning. The patient was referred to a tertiary center for an elective surgical repair. Finally, we provided a detailed review of the literature concerning the intermediate type of atrioventricular septal defect.

**Conclusion:**

Although 2D transthoracic and transesophageal echocardiography enables diagnosis of the intermediate type atrioventricular septal defect, precise assessment of anatomy of atrioventricular septal defects and common atrioventricular valve was enabled only by real time 3D echocardiography.

## Introduction

Intermediate atrioventricular septal defect (AVSD) is defined as malformation lying between persistent ostium primum with cleft in the superior bridging leaflet and the complete form of common atrioventricular (AV) orifice [1]. Intermediate type AVSD is less frequent than complete or partial AVSD, and is rarely encountered in the elderly and the utility of three dimensional transesophageal echocardiography (3D-TEE) in the diagnosis of intermediate AVSD has not been reported to date. Here, we report an adult case of intermediate AVSD, where for the diagnosis 3D-TEE was very useful, and a literature review of all the adult cases of intermediate AVSD that were reported.

## Case report

A 52 year old female patient with a shortness of breath (New York Heart Association class III) and vertigo on exertion, gradually worsening exercise intolerance and occasionally chest pain for the last 4 months was referred to the Eastern Slovakian Institute of Cardiovascular Diseases for evaluation. Although as a child she was diagnosed with a murmur, she had not been treated and she was lost from the follow-up. At presentation she had a blood pressure of 110/95 mmHg and permanent atrial fibrillation. She exhibited a 1/6 systolic murmur and electrocardiogram showed right axis deviation and signs of right ventricular overload. On physical examination, peripheral edema was present. Serum NTpro brain natriuretic peptide was elevated, 2324 pg/ml.

**Fig. 1:**
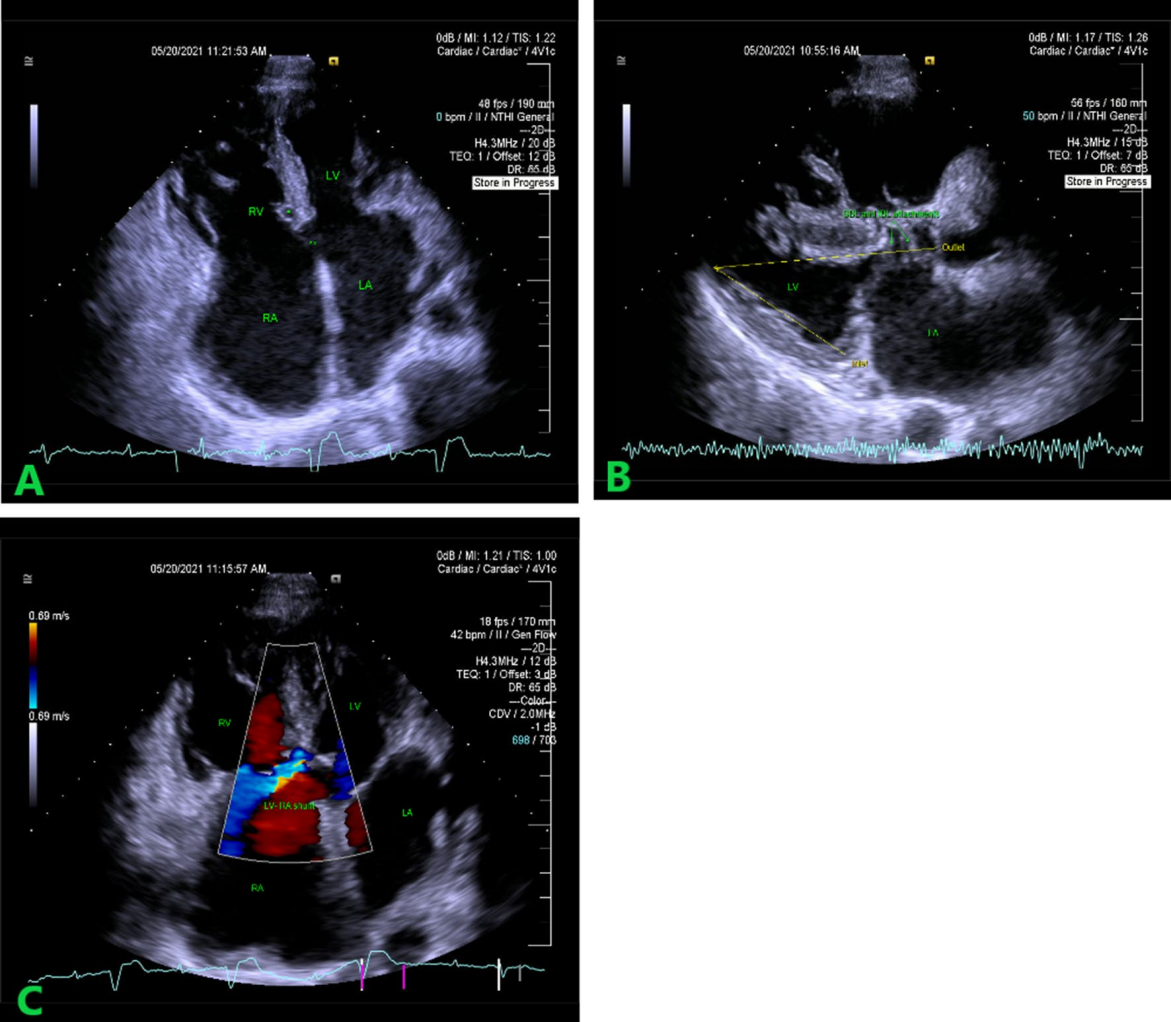
**A** Atrioventricular septal defect. 2D TTE—apical four chamber view. AV septum deficiency (double-asterisk). Right heart chambers enlargement. Both AV valves are placed at the same level. The tricuspid pouch (asterisk). **B** Left ventricular outflow tract prolongation. Parasternal long axis view. Larger outlet dimension compared to the inlet portion distance. Hemodynamically insignificant obstruction of LVOT, caused by the attachment of the bridging leaflets. **C** Left ventricle to right atrium shunt due to AV septum insufficiency. Color flow-mapping imaging. *TTE* transthoracic echocardiography, *AV* atrioventricular, *LVOT* left ventricular outflow tract

Two-dimensional transthoracic echocardiography with a CW Doppler (Siemens SC 2000 Prime, Siemens Medical Solutions, Inc, Mountain View, California, USA) (2D-TTE) revealed AV septum deficiency with enlargement of the right heart chambers (right ventricle and right atrium) and hemodynamically significant left-to-right shunt (pulmonary to systemic blood flow 2:1), right and left AVV valves located at the same level, with bridging leaflet chordal attachments to the crest of the interventricular septum (IVS), typically seen in AVS, right ventricle ejection fraction slightly decreased, severe valvular regurgitation of the common AV valve, large septum primum defect (23 × 28 mm), defect in the membraneous part of the IVS and left ventricle (LV) to right atrium shunt. Moreover, left ventricular outflow tract was prolonged with a hemodynamically insignificant obstruction caused by the attachment of the bridging leaflets. Left ventricle was of normal size with normal global systolic function (Fig. 1A–C).

**Fig. 2:**
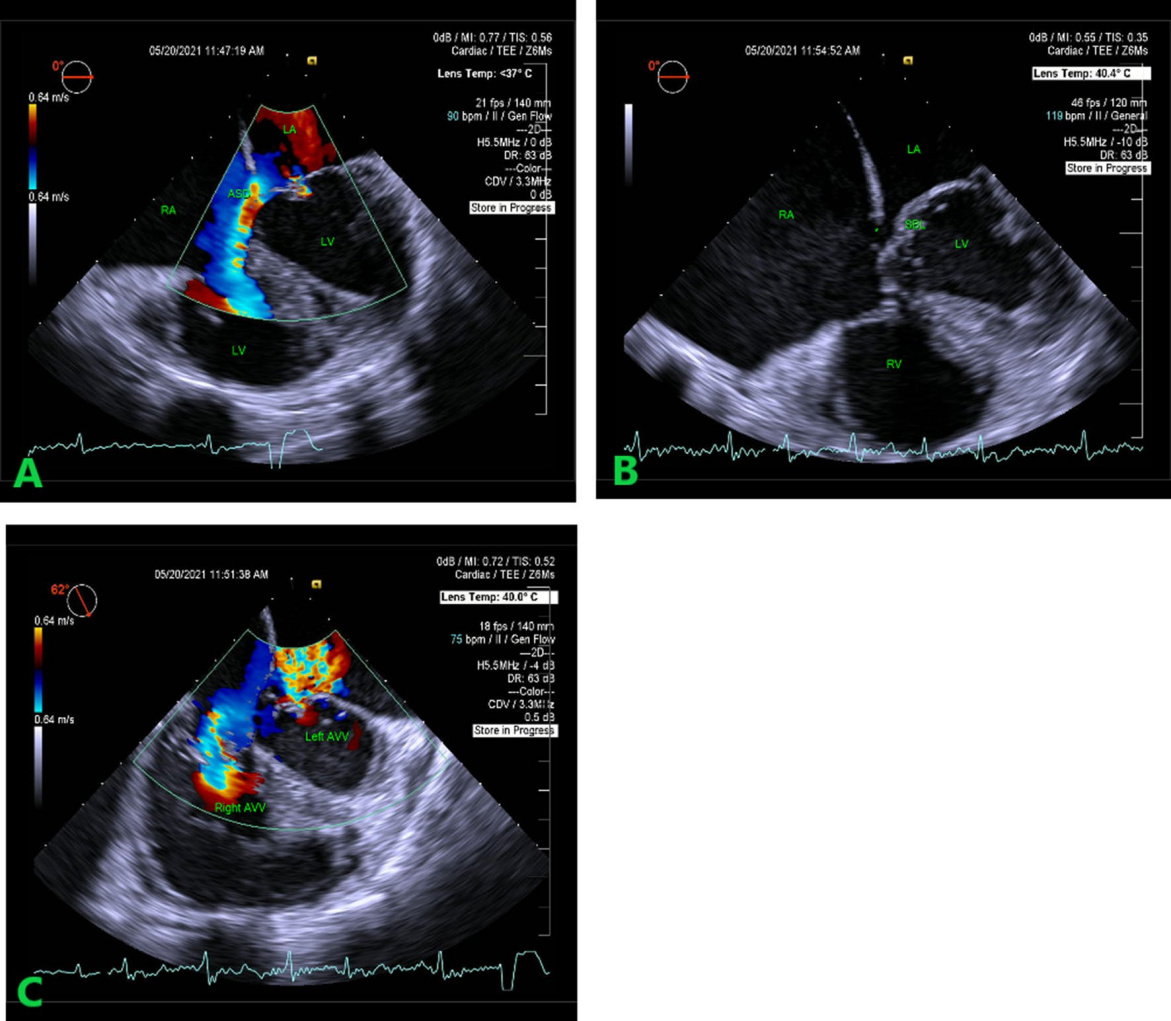
**A** AVSD—2D TEE. Primum atrial septal defect with a left-to-right atrial shunt in the color flow mapping. **B** 2D-TEE. AV septum deficiency and superior bridging leaflet attachment to the interventricular septum. Midesophageal four-chamber view. The primum atrial septal defect is also seen (asterisk). **C** AV valves regurgitation. Color flow-mapping imaging of the common AV valve presented as severe valvular regurgitation with a higher grade observed on the right component. *AVSD* atrioventricular septal defect, *TEE* transesophageal echocardiography, *AV* atrioventricular

Transesophageal 2D echocardiography (TEE) with a continuous wave (CW) Doppler, confirmed TTE findings of AV septum deficiency, primum atrial septal defect with left-to-right shunt with better visualization of superior bridging leaflets attachments to the IVS and AVV regurgitation (Fig. 2A–C).

According to the above results of the 2D echocardiography, the diagnosis of AVSD intermediate (transitional) type was made. For precise assessment of AV septal defects and common AV valve anatomy real time three-dimensional echocardiography (RT3D) was performed.

**Fig. 3:**
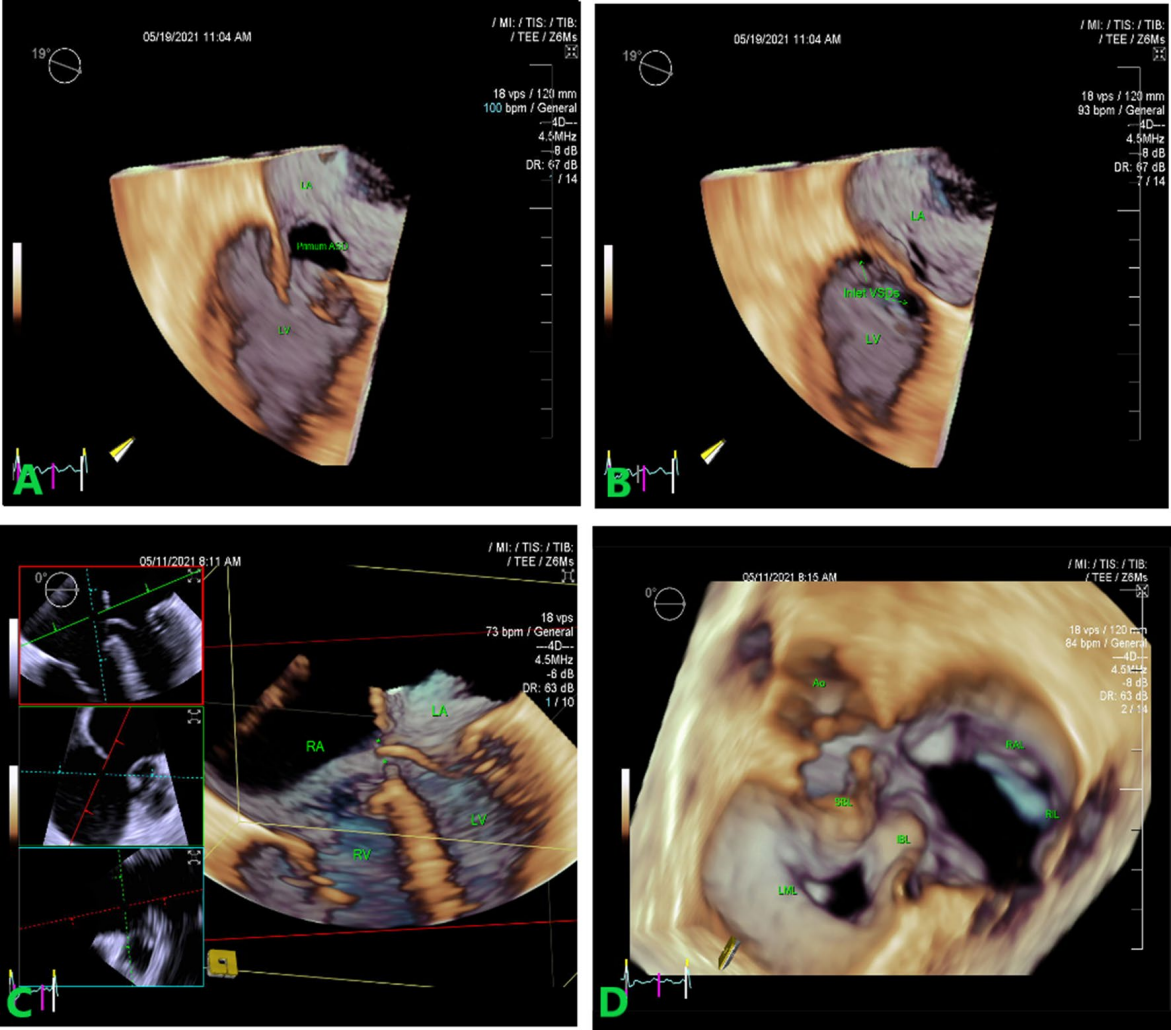
**A** AVSD—AV septum deficiency. 3D TEE. 3D high volume, high resolution, and single-beat sample, cropped form the front using the green plane adjustment reveals the AV septum deficiency (asterisk). **B** 3D echocardiography of the primum atrial septal defect—left sided en face view. **C** Inlet VSDs. LV en face view of inlet interventricular septal defects. Smaller defects (asterisks) in membranous part of IVS. **D** RT3D TEE left atrial perspective view of the common atrioventricular valve. The common AV valve is with one annulus and two orifices. Both the superior and inferior bridging leaflets are seen. Triangle appearance of the left sided orifice and the elliptic shape of right sided AV orifice. The common valve consist of five leaflets—superior bridging leaflet (SBL), inferior bridging leaflet (IBL), left mural leaflet (LML), right mural or inferior leaflet (RML) and right anterior or superior leaflet (RAL). *AVSD* atrioventricular septal defect, *TEE* transesophageal echocardiography, *VSD* ventricular septal defect, *AV* atrioventricular

Real time 3D transthoracic echocardiography (RT3DTTE) enabled complete 3D analysis of the right ventricle, with a calculated ejection fraction of 34% and end-diastolic volume of 161 ml. Real time 3D transesophageal echocardiography (RT3DTEE) revealed an oval in appearance atrial septal primum defect, which could not be visualized in 2D echocardiography with a more precise measurements of 31 × 23 mm. Moreover, two small inlet VSD in the membraneous part of the IVS, between the chordate attachements to the crest of IVS of bridging leaflets, with sizes of 4 × 5 and 7 × 8 mm with a left-to-right shunt were also detected. The common AV valve was found to be with one annulus and two orifices different in shape (triangle on the left side, elliptic on the right side) with malposition of the aorta. The common valve was found to have five leaflets-superior, inferior, left mural, larger right anterior or superior and smaller right inferior leaflet. The left mural leaflet was relatively small with slight restriction. Finally, no tricuspid pouch, described in 2D TTE was detected (Fig. 3A–D).

**Fig. 4:**
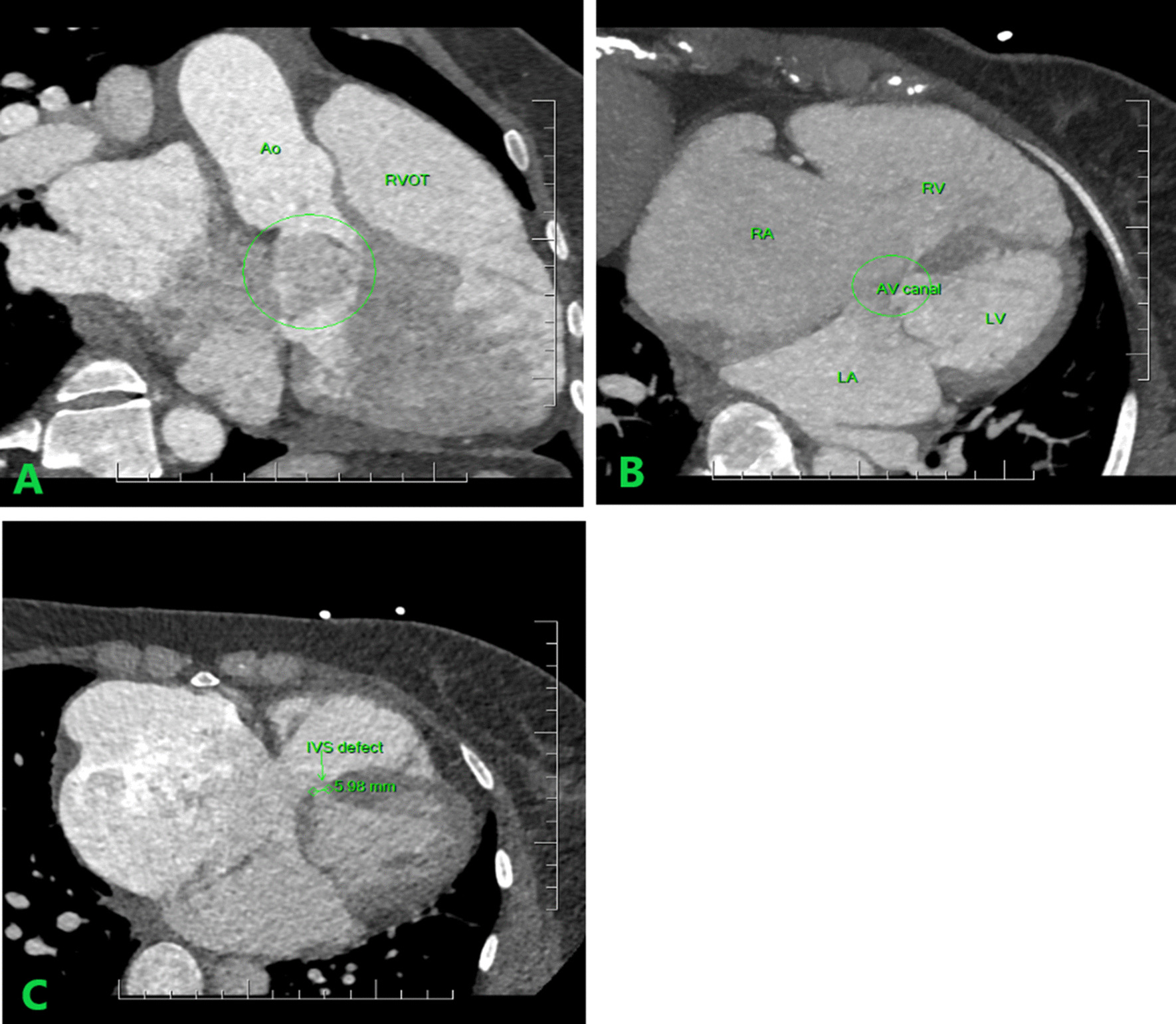
**A** Atrioventricular septal defect. Four-chamber CCT image showing the open atrioventricular canal valve, **B** four-chamber CCT image showing the atrioventricular canal, **C** four-chamber CCT image showing the intraventricular septal defect of 6 mm. *CCT* cardiac computed tomography

The main findings of the cardiac computed tomography (CCT) were, intermediate AVSD, large atrial septal defect (17 mm), smaller membraneous ventricular septal defect (6 mm), extremely dilated right heart chambers, mainly the right atrium and dilated left atrium, large communication between the atrial septal defect and the right atrium, common AV valve, with the tricuspid part attached to the free wall and the the mitral part (anterior leaflet) thickened and attached to the interventricular septum (Fig. 4A–C).

Right heart catheterization showed bidirectional shunt (1:1) at the level of the left atrium and right ventricle, mild pulmonary hypertension (22 mmHg), and pulmonary vascular resistance of 1.7 Wood Units. Left heart catheterization showed no coronary disease.

Initially, due to the lack of expertise by the local hospital, the patient was considered by the local heart team as highly risk (right ventricular dysfunction and dilatation) for surgical correction, and she was offered only conservative treatment. Later, the indication was re-evaluated and she was referred to the National Institute of Cardiovascular Diseases for an elective surgery. The National Institute is a tertiary hospital with an expertise in correction of congenital heart diseases and especially complex patients like the patient who is discussed in this case report.

## Discussion

Atrioventricular septal defect is a complex cardiac anomaly, the status of which varies according to the type of the disease. Patients with complete or intermediate type AVSD rarely survive decades without appropriate treatment and prolong survival is exceptional. There have been no reports regarding the natural history of intermediate type AVSD. However, considering the long-term survival of other types of AVSD (i.e. complete common AVSD 30–73 years) [2, 3]. Tandon et al. [4] reviewed 139 patients with AVSD, and only 5 patients (complete AVSD 1, intermediate type AVSD 1, partial AVSD survived to 46 years of age or older. Here we report, a 52 years old patient with intermediate type AVSD, therefore our patient survived for an unusual long time.

The anatomy of intermediate type AVSD varies widely, and the surgical approach is not uniform but must be modified in each case. Bharati et al. [1] classified the disease into three types based on AV valve morphology: (1) type I, which is two separate AV valves with cleft, (2) type II, which is AV orifice divided by AV valve tissue or summit of VSD, or both, and (3) type III, which is anterior and posterior bridging leaflets similar to complete AVSD. The present case was similar to type II, with separation of the AV valve orifice by AV valve tissue and the VSD crest.

There are few reports in the literature concerning the intermediate type of AVSD. The first case of intermediate type of AVSD was reported, was by Tandon et al. [4], and it was about a female patient who died at the age of 65 years old and the diagnosis of the intermediate type of AVSD was confirmed during the pathologic examination. Later another two cases of intermediate type of AVSD were reported by Honda et al. [5] and Matsumoto et al. [6], where in a 51 year old male patient and an 82 years old female patient respectively, the intermediate type of AVSD were confirmed also during autopsy. The first adult case of intermediate type of AVSD in Japan successfully operated, was reported by Ataka et al. [7]. It was about a 42 years old female patient that she underwent patch closure of the ostium primum defect (ASD) and mitral and tricuspid valvuloplasty. Tatebe et al. [8] reported the case report of a 65 years old woman with intermediate type of AVSD that underwent surgical repair. The surgery included direct closure of the ventricular septal defect (VSD), repair of the cleft in the atrioventricular valve, and ostiun primum closure. Moreover, in a series of 10 patients [mean age 3.3 years (0.1–33)] with AVSD, Mace et al. [9] reported a 26 years old patients that underwent successful intermediate AVSD repair with a surgically created double-orifice left atrioventricular valve.

Erdemli et al. [10] also reported a 45 years old female patient that underwent atrial septal defect closure with a patch, direct ventricular septal defect closure and commissural valvuloplasty of the double-orifice atrioventricular valve due to intermediate type AVSD. Miyamoto et al. [11] reported the utility of the 3D- transthoracic echocardiography (3D-TTE) in the diagnosis of an intermediate type of AVSD in a 54 years old patient. Moreover, Gao et al. [12] reported a totally robotic repair (ASD patch closure, direct VSD closure, mitral valve cleft repair) of AVSD in a 24 years old lady who was diagnosed with an intermediate AVSD. Finally, De Angelis et al. [13] reported the case of a 50 years old male patients with intermediate AVSD who was referred to reparative surgery.

Except from the three autopsy cases that were described in the literature review [4–6], 2D-transthoracic echocardiography (2D-TTE), 2D-TEE, color Doppler echocardiography, right heart catheterization, and coronary angiography were the main imaging and diagnostic modalities that were used in the rest of the cases [7–13] for the diagnosis and evaluation of the intermediate AVSD. Although complete 2D echocardiography combined with 2D TEE enables diagnosis of intermediate type of AVSD, complete assessment of such pathology is possible only by using RT3D echocardiography.

Miyamoto et al. [11] reported the utility of 3D-TTE in the diagnosis of intermediate type AVSD. Intermediate AVSD has many variable components, and visualization of the components is important for selecting the definitive treatment. In 3D echocardiography atrial septal defect could have been evaluated and measured precisely, comparing to 2D echocardiography, were it was not observed. The size and appearance of the atrial septal defect is key because this is partly responsible for the right ventricular dilatation.

Similarly, in 3D echocardiography compared to 2D imaging, inlets of the ventricular septal defects were clearly seen with small jets between dense chordae of both bridging leaflets, which is typically seen in intermediate type of AVSD. In 2D echocardiography, an IVS pouch finding is also typically seen in AV septal defect, as nicely described Miyamoto et al. [11] however, we did not confirm the presence of it, neither in 3D TTE or 3D TEE, so we suppose that in 2D TTE finding resembles to pouch was only an artefact although LV to RA shunt characterized defect in membranous part of interventricular septum was present.

Two separate orifices with one annulus is another sign of this type of AVSD. Besides that, additional features of AVSD such as prolongation of the LVOT or bridging of both leaflets over the crest of IVS were observed as well. Collectively, 3D-TTE provides additional important information, which is not always available with the use of 2D-TTE, for surgical treatments.

In our case report we additionally used for the diagnosis and evaluation of the intermediate AVSD real time 3D-TEE. This imaging modality for the diagnosis of intermediate AVSD is not enough reported and described in the literature. Faletra et al. [14], described the use of real time 3D-TEE for the diagnosis of a complete AVSD.

According to the current European Society of Cardiology guidelines for the management of adult congenital heart disease (15) the patient described above is indicated for surgical repair of the AVSD, and this was the reason why the initial indication for conservative treatment of the local heart team was re-evaluated and the patient was referred to the National Institute of Cardiovascular Diseases for surgery. In our opinion, these complex congenital heart diseases cases should only be referred to tertiary centers where they have the expertise to deal with such patients.

In conclusion, we described a rare case of intermediate AVSD in an adult patient and we showed the valuable utility of real time 3D-TEE. Although 2D TTE and TEE enables diagnosis of intermediate type AV septal defect, precise assessment of anatomy of AV septal defects and common AV valve was enabled only by RT3D echocardiography. RT3D echocardiography provides very quick imaging modality for precise description not only about anatomic localization of the defect, but also about AV chordae attachment, which are the clue information before surgical decision making and future surgical planning.

## Data Availability

The datasets used and/or analyzed during the current study are available from the corresponding author on request.

## Abbreviations

AVSD: Atrioventricular septal defect
AV: Atrioventricular
IVS: Interventricular septum
VSD: Ventricular septal defect
ASD: Atrial septal defect
3D-TEE: Three dimensional transesophageal echocardiography
CW: Continuous wave
2D-TTE: Two dimensional transthoracic echocardiography
RT3DTTE: Real time three dimensional transthoracic echocardiography
RT3DTEE: Real time three dimensional trasesophageal echocardiography
CCT: Cardiac computed tomography
LVOT: Left ventricular outflow tract,
LV: Left ventricular
RA: Right atrium
